# *mucG, mucH,* and *mucI* Modulate Production of Mutanocyclin and Reutericyclins in Streptococcus mutans B04Sm5

**DOI:** 10.1128/jb.00042-22

**Published:** 2022-04-11

**Authors:** Jonathon L. Baker, Xiaoyu Tang, Sandra LaBonte, Carla Uranga, Anna Edlund

**Affiliations:** a Genomic Medicine Group, J. Craig Venter Institute, La Jolla, California, USA; b Department of Pediatrics, University of California at San Diego, La Jolla, California, USA; c Institute of Chemical Biology, Shenzhen Bay Laboratory, Shenzhen, China; d Department of Biochemistry & Biophysics, Texas A&M University, College Station, Texas, USA; Ohio State University

**Keywords:** *Streptococcus mutans*, dental caries, oral microbiome, reutericyclin, biosynthetic gene cluster, mutanocyclin

## Abstract

Streptococcus mutans is considered a primary etiologic agent of dental caries, which is the most common chronic infectious disease worldwide. S. mutans B04Sm5 was recently shown to produce reutericyclins and mutanocyclin through the *muc* biosynthetic gene cluster and to utilize reutericyclins to inhibit the growth of neighboring commensal streptococci. In this study, examination of S. mutans and *muc* phylogeny suggested evolution of an ancestral S. mutans *muc* into three lineages within one S. mutans clade and then horizontal transfer of *muc* to other S. mutans clades. The roles of the mucG and mucH transcriptional regulators and the mucI transporter were also examined. *mucH* was demonstrated to encode a transcriptional activator of *muc*. *mucH* deletion reduced production of mutanocyclin and reutericyclins and eliminated the impaired growth and inhibition of neighboring streptococci phenotypes, which are associated with reutericyclin production. Δ*mucG* had increased mutanocyclin and reutericyclin production, which impaired growth and increased the ability to inhibit neighboring streptococci. However, deletion of *mucG* also caused reduced expression of *mucD*, *mucE*, and *mucI.* Deletion of *mucI* reduced mutanocyclin and reutericylin production but enhanced growth, suggesting that *mucI* may not transport reutericyclin as its homolog does in Limosilactobacillus reuteri. Further research is needed to determine the roles of *mucG* and *mucI* and to identify any cofactors affecting the activity of the *mucG* and *mucH* regulators. Overall, this study provided pangenome and phylogenetic analyses that serve as a resource for S. mutans research and began elucidation of the regulation of reutericyclins and mutanocyclin production in S. mutans.

**IMPORTANCE** S. mutans must be able to outcompete neighboring organisms in its ecological niche in order to cause dental caries. S. mutans B04Sm5 inhibited the growth of neighboring commensal streptococci through production of reutericyclins via the *muc* biosynthetic gene cluster. In this study, an S. mutans pangenome database and updated phylogenetic tree were generated that will serve as valuable resources for the S. mutans research community and that provide insights into the carriage and evolution of S. mutans *muc.* The MucG and MucH regulators, and the MucI transporter, were shown to modulate production of reutericyclins and mutanocyclin. These genes also affected the ability of S. mutans to inhibit neighboring commensals, suggesting that they may play a role in S. mutans virulence.

## INTRODUCTION

Streptococcus mutans is considered a primary etiologic agent of dental caries, which is the most common chronic infectious disease worldwide (1). As it is not typically considered a pioneer colonizer of the tooth surface, S. mutans must be able to outcompete already established bacterial neighbors (which are typically health-associated commensals) to successfully establish itself as a member of the dental plaque microbiota and cause disease (2, 3). To achieve this outcome, S. mutans uses several different abilities. S. mutans is able to generate insoluble glucans from sucrose, which greatly facilitate biofilm formation on the tooth surface (4). Therefore, in the presence of a carbohydrate-rich diet (particularly one with frequent consumption of sucrose), S. mutans is at a distinct advantage compared to many of its more health-associated neighbors (2, 3). In addition, S. mutans utilizes these host dietary carbohydrates in energy metabolism. This process generates organic acids that quickly and drastically lower the local pH, which is what causes damage to the underlying tooth (1). While S. mutans employs a complex and robust acid tolerance response to continue to thrive in these acidic conditions, many of its health-associated competitors are much more acid-sensitive and cannot sustain growth (5). In addition to these somewhat indirect competition strategies, S. mutans also directly inhibits the growth of its competitors through the production of antimicrobial small molecules, such as bacteriocins (which are termed mutacins in S. mutans) (6).

One group of these antimicrobial small molecules are reutericyclins, which are acylated tetramic acids produced by a biosynthetic gene cluster (BGC), *muc* (Fig. S1), encoded by a subset of globally distributed S. mutans strains (7–9). The *muc* BGC consists of 10 genes (*mucA* to *mucJ*) (8, 9). *mucD* and *mucE* encode the biosynthetic core proteins: a nonribosomal peptide synthetase (NRPS) and a polyketide synthase (PKS), respectively. *mucA*, *mucB*, and *mucC* are predicted to encode tailoring enzymes (8). *mucF* was recently shown to encode a previously unknown class of acylase with an HXXEE motif, while *mucG* and *mucH* are predicted to encode TetR/AcrR family transcription regulators. *mucI* is predicted to encode a DHA2 family transporter, and *mucJ* is predicted to encode a small multidrug export protein (8, 9). In S. mutans B04Sm5, *muc* produces four tetramic acid compounds: three reutericyclin molecules (differing in the length and saturation of the acyl chain, and referred to collectively in this paper simply as “reutericyclin”) and the unacylated tetramic acid, mutanocyclin (8). Previous experiments illustrated that deletion of the gene encoding the MucF acylase abolishes production of unacylated mutanocyclin (8). As a result, the Δ*mucF* strain accumulates more reutericyclin molecules, which leads to growth inhibition of both itself and neighboring health-associated oral streptococci (8). Recently, reutericyclin treatment was shown to inhibit biofilm formation and acid production by an *in vitro* oral microbiome community, as well as significantly alter the taxonomic profile of the community (10). Meanwhile, mutanocyclin did not have any antimicrobial activity against several species of oral streptococci but demonstrated anti-inflammatory activity in a murine model (9). Mutanocyclin treatment did not alter the taxonomic profile of an *in vitro* oral biofilm community to the same extent as reutericyclin, but did significantly reduce the abundance of Limosilactobacillus fermentum, specifically (10). Other possible roles of mutanocyclin on S. mutans metabolism and ecology, including whether mutanocyclin has direct bactericidal or bacteriostatic activity against L. fermentum, are the subject of current investigation. As the majority of research on S. mutans has been conducted using well established type strains that do not encode *muc* (such as UA159 and UA140) the roles of *muc*, and its products, within the S. mutans lifestyle are not well understood.

In this study, the complete genome of B04Sm5, which was recently reported (11), was analyzed, and pangenome analysis of 244 S. mutans genomes from NCBI was performed to examine the distribution of the genes within the *muc* BGC. Additionally, the roles of *mucG*, *mucH*, and *mucI* vis-à-vis the production of mutanocyclin and reutericyclin, regulation of *muc*, overall transcriptome, and ability of S. mutans to inhibit neighboring commensals were further investigated.

## RESULT

### Comparative genomics of B04Sm5, the S. mutans pangenome, and distribution of *muc*.

**FIG 1:**
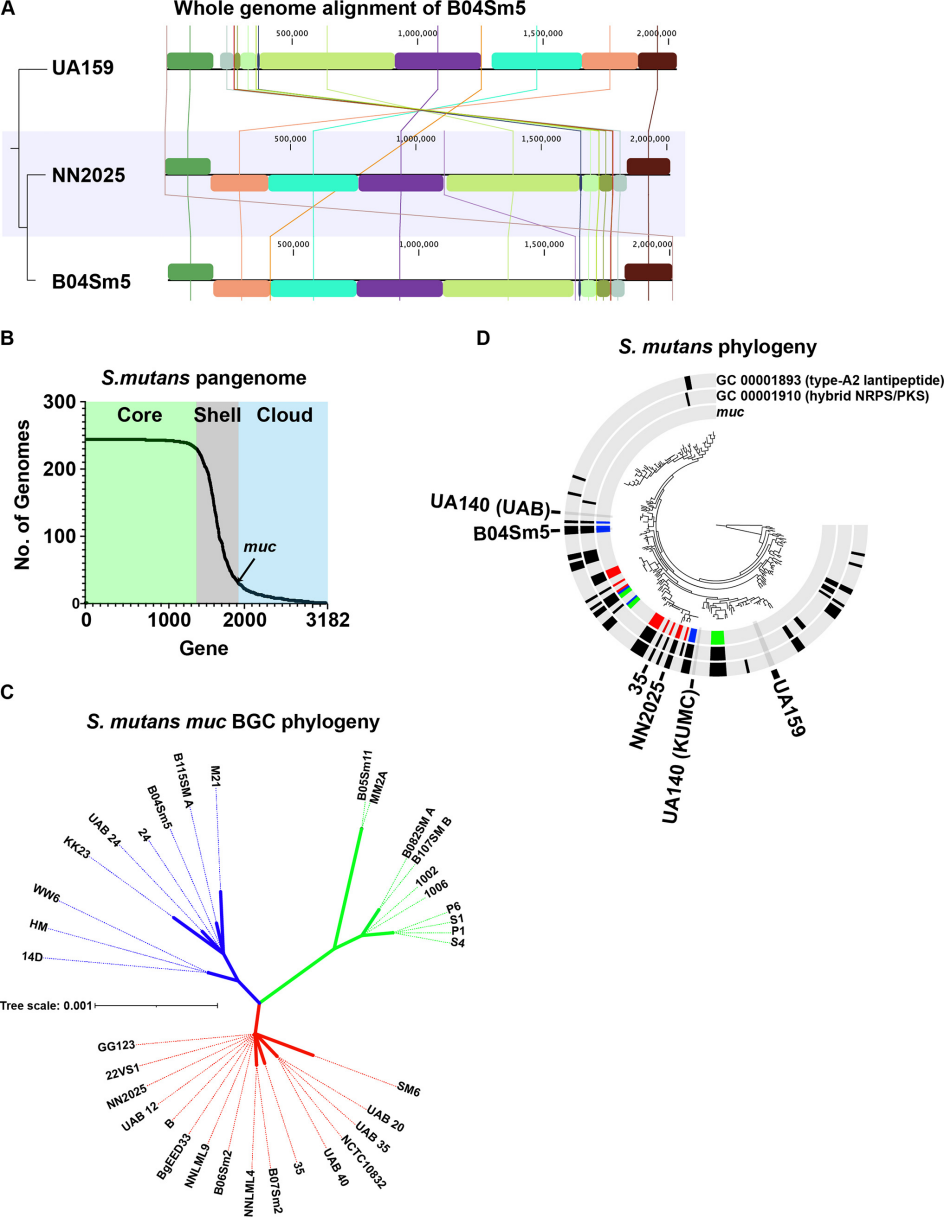
Comparative genomics of B04Sm5 and the pangenome of S. mutans. (A) Whole-genome alignment of S. mutans B04Sm5 versus the type strains UA159 (*− muc*) and NN2025 (+ *muc*). The tree on the left is based on the whole-genome alignment itself. (B) The S. mutans pangenome. Graph showing the gene clusters in the S. mutans pangenome versus the number of genomes that each gene appears in. The core (>90% of genomes), shell (≥10% and ≤90% of genomes), and cloud (<10% of genomes) pangenomes are indicated by the background colors green, gray, and blue, respectively. The location of *muc* on the graph is indicated by the arrow. (C) S. mutans *muc* biosynthetic gene cluster (BGC) phylogeny. Concatenated protein sequences of MucA-I across the 35 S. mutans genomes harboring *muc* were used to construct an unrooted phylogenetic tree of the gene cluster. The three main lineages are colored blue, green, and red. (D) S. mutans phylogeny. Phylogenetic tree of 244 S. mutans genomes based on the concatentated protein sequences of 12 core genes, as described under Materials and Methods. The tree is annotated with three layers indicating the presence of *muc*, GC0000189 (type A2 lanthipeptide, which was correlated with muc in Fig. S2) and GC00001910 (hybrid nonribosomal peptide synthetase / polyketide synthase [NRPS/PKS], which was correlated with *muc* in Fig. S2). The presence/absence bars in the *muc* layer are colored based on the position of the *muc* BGC of the cognate genome in the *muc* phylogenetic tree in panel C. S. mutans genomes of interest are labeled (B04Sm5, UA159, UA140-KUMC, UA140-UAB, NN20205, and 35). The full phylogenetic tree, with all leaves labeled, is available in Fig. S3.

The complete genome sequence of S. mutans B04Sm5, the strain in which reutericyclin and mutanocyclin production was recently elucidated, has now been reported (11). Compared to the S. mutans type strain, UA159, B04Sm5 has a large ∼1.4 Mbp X-shaped chromosomal inversion, as did NN2025, another S. mutans isolate with a published complete genome sequence (12), which also encodes *muc* (Fig. 1A). To examine the distribution of the genes of the *muc* BGC across the S. mutans pangenome, the genomes of 244 S. mutans strains from GenBank (all S. mutans genomes on GenBank, as of June 2021; Table S1) were examined using anvi'o (13). Using the parameters described under Materials and Methods, the S. mutans pangenome included 3,183 genes. The pangenome data table file was too large to be included as Supplemental Material, but is publicly available at https://github.com/jonbakerlab/Smutans-pangenome. There were 1,421 genes in the core genome (present in >90%, or 232, of the genomes), 1,212 genes in the cloud pangenome (present in <10%, or 36 of the genomes), and 549 genes in the shell pangenome (present in ≥10% and ≤90% of the genomes) (Fig. 1B). *muc* was found on the boundary of the cloud and shell pangenomes, present in 35 of the 244 genomes, with all genes in the BGC (anvi'o gene calling did not identify *mucJ*, but a subsequent manual search did) present in all 35 of these strains, indicating there were no versions of the *muc* BGC in S. mutans missing any of the 10 genes (https://github.com/jonbakerlab/Smutans-pangenome). Concatenated protein sequences of MucA-I were used to construct a phylogenetic tree of the *muc* BGC within S. mutans, which demonstrated three main lineages of Muc (Fig. 1C). To search for other genes that may affect whether it is advantageous for S. mutans to encode *muc*, the pangenome was examined for genes that co-occurred with the *muc* BGC using Coinfinder (14). A total of 44 genes had their carriage correlated with that of *muc* (Fig. S2; Table S2). Of those 44 genes associated with *muc*, 28 were found in B04Sm5; however, none were located adjacent to *muc* in the chromosome. Notable among the correlated genes were another hybrid NRPS/PKS BGC (GC00001910) and a lantipeptide BGC (GC00001893). No genes were significantly negatively correlated with carriage of *muc*, using the parameters employed, indicating that there may be no genes in the S. mutans pangenome that contraindicate carriage of *muc* or the production of reutericyclin or mutanocyclin.

To further examine phylogeny of S. mutans and distribution of *muc*, the pangenome was used to identify optimal protein sequences to use in constructing a phylogenetic tree of the 244 S. mutans strains. The pangenomics analysis identified 348 single-copy core genes encoded by all 244 genomes. Using an approach described by Eren et al. (13), these genes were filtered by a minimum geometric homogeneity index of 1 to remove protein sequences that would introduce gaps in the alignment, which left 270 protein sequences. Many of these genes had nearly identical sequences; therefore, a further filter of maximum functional homogeneity index was set to 0.9925, leaving 12 genes (Table S3) on which to base the phylogenetic analysis (Fig. 1D; Fig. S3). The strains encoding *muc* were not monophyletic, and in some cases, the three main clades of *muc* did not line up with overall S. mutans phylogeny. These results suggest horizontal transfer of *muc* between S. mutans clades, as has been suggested for Lactobacillus spp. carrying the closely related reutericyclin BGC (15). Note that there are two assemblies on NCBI RefSeq both described as “S. mutans strain UA140” deposited by different groups: GCF_008831365.1, a complete genome, and GCF_012641085.1, which is fragmented into 21 contigs. The sequences of the assemblies are substantially different, as indicated by our phylogenetic analysis (Fig. 1D; Fig. S3). This discrepancy in the sequences of a widely used type strain highlights the need for researchers to sequence their lab strains to ensure experimental consistency and proper nomenclature across the field.

In terms of arsenal of small molecules produced by BGCs other than *muc*, the B04Sm5 genome encodes the type A2 lanthipeptide bacteriocin mentioned above (found in 51 genomes across S. mutans, not in UA159), mutacin IV/nonlanthibiotic mutacin *nlmAB* (found in 136 S. mutans genomes) (16–18), mutacin V/*cipB* (various genes found in 91 to 243 S. mutans genomes) (16, 17), mutacin VI/*nlmD* (found in 229 S. mutans genomes) (19, 20), as well as the *nlmTE* transporter to export these nonlanthibiotic mutacins (present in all 244 S. mutans genomes) (21) (Table S3). B04Sm5 does not encode the BGC for mutanobactin (22), which is present in ∼95 genomes, including UA159, or the ribosomally synthesized and post-translationally modified peptide RaS-RIPP BGC (*cidAB*) (23), which is found in UA159, UA140, and NN2025 (18) (and 181 S. mutans genomes in total). These gene clusters of interest are listed in Table S3.

### Deletion of *mucG*, *mucH*, or *mucI* affects production of mutanocyclin and reutericyclin.

**FIG 2:**
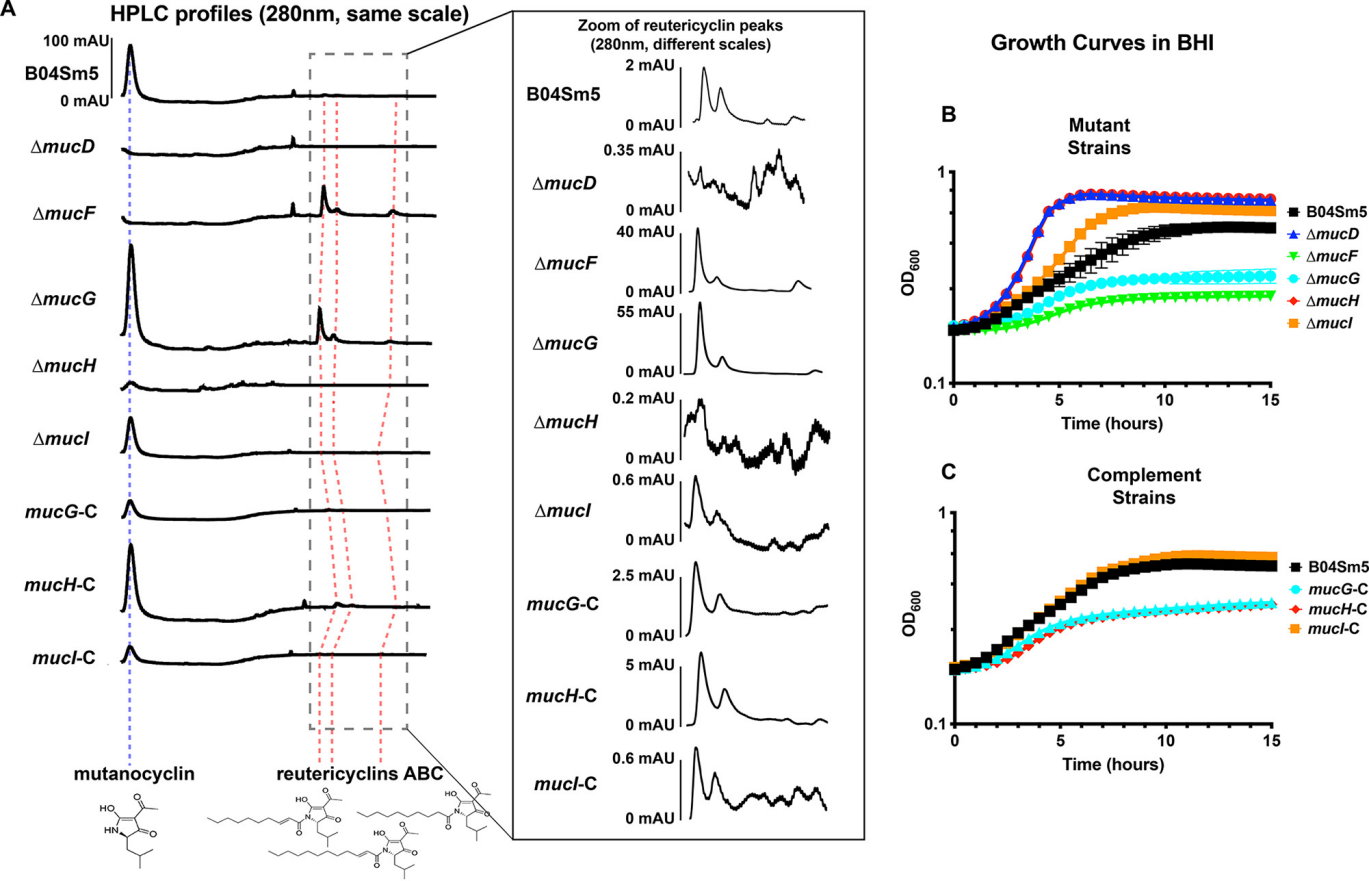
Deletion of the *mucG*, *mucH*, or *mucI* affects production of mutanocyclin and reutericyclins and growth of B04Sm5. (A) High-performance liquid chromatography (HPLC) profiles of concentrated extracts from the supernatant of the indicated strain. Profiles are cropped to retention times of 17 to 31 min, aligned to the mutanocyclin peak (indicated by the dashed blue line), and presented on the same scale. Red dashed lines indicate the peaks of reutericyclins A, B, and C. The inset shows a zoom of retention times of 27 to 31 min to better illustrate the reutericyclin peaks. Note that unlike the main panel, the profiles in the inset are not to the same scale, and the profile of each strain has a scale bar with the scale of the peaks for reference. (B, C) Growth of the parent strain B04Sm5 and its Δ*mucD*, *ΔmucF*, *ΔmucG*, *ΔmucH*, and Δ*mucI* derivatives (B) or complement strains *mucG*-C, *mucH*-C, and *mucI*-C (C) in brain heart infusion (BHI) (*n* = 8 for each strain). OD_600_, optical density at 600 nm.

To examine the roles of the predicted TetR/AcrR family transcriptional regulators within the *muc* BGC, *mucG*, and *mucH*, as well as the DHA2-like family transporter, *mucI*, single-gene deletion mutants were generated as described under Materials and Methods. To illustrate that any phenotypes observed in the mutant strains were not due to polar effects, complement strains (*mucG*-C, *mucH*-C, and *mucI*-C) were produced where the gene of interest was reinserted in a distant locus, as described under Materials and Methods. The deletion mutants Δ*mucD* and Δ*mucF* were described previously (8). Cultures of these strains, as well as Δ*mucD*, Δ*mucF*, and the parent strain B04Sm5, were analyzed by high-performance liquid chromatography (HPLC) to observe production of mutanocyclin and the reutericyclins. As seen in the previous study (8), deletion of *mucD* abolished production of all four tetramic acids produced by *muc*, while deletion of *mucF* eliminated production of the unacylated mutanocyclin and increased production of the acylated reutericyclins (Fig. 2A). Compared to B04Sm5, deletion of *mucG* increased production of both mutanocyclin and the reutericyclins, while deletion of *mucH* or *mucI* decreased production of both mutanocyclin and the reutericyclins, with Δ*mucH* having lower production than Δ*mucI* (Fig. 2A). Complementation with *mucG* and *mucH* reversed the phenotype observed in the cognate mutant, indicating that the phenotypes observed were true effects of the deletion of *mucG* or *mucH* and not simply polar disruptions in the expression of the adjacent genes (Fig. 2A). In fact, the phenotypes were reversed to a greater extent than was observed for B04Sm5 (i.e., *mucG-*C had lower production of the tetramic acids than B04Sm5, while *mucH*-C had higher production than B04Sm5) (Fig. 2A). This may indicate that expression of *mucG* and *mucH* may be increased in the complement strains, where transcription is driven by the *gtfA* promoter, compared to transcription driven by the cognate native loci promoters in B04Sm5. Meanwhile, both Δ*mucI* and *mucI-*C had reduced tetramic acid production compared to B04Sm5 (Fig. 2A). Several smaller peaks between the mutanocyclin and reutericyclin peaks can be discerned across the strains to various degrees, and were observed previously by Tang et al. (8); however, their identity or possible significance is not currently known. Since it is supernatants, and not cell lysate, being analyzed here, it is possible that MucI exports reutericyclin and/or mutanocyclin, as reflected by the smaller amount detected in the supernatant of the Δ*mucI* strain.

### Deletion of *mucG* results in impaired growth, while deletion of *mucH* or *mucI* results in improved growth.

Growth curves of the Δ*mucD*, Δ*mucF*, Δ*mucG*, and Δ*mucH* strains, as well as the complement strains, and the parent strain, B04Sm5, were performed in brain heart infusion (BHI) media. Deletion of *mucG* resulted in significantly impaired growth, nearly to the same level as the Δ*mucF* strain (Fig. 2B). Meanwhile, deletion of *mucH* or *mucI* resulted in increased growth, reminiscent of the Δ*mucD* strain (Fig. 2B). Complementation of all three genes reversed the phenotypes observed in the cognate mutant strain (Fig. 2C). While the *mucI*-C strain restored a growth rate similar to the B04Sm5 parent strain, the mucH-C strain actually decreased growth much further, nearly to the level of the Δ*mucG* strain (Fig. 2C). The mucG-C strain had improved growth compared to Δ*mucG* but still displayed impaired growth compared to B04Sm5 (Fig. 2C). Collectively, these results are in line with the HPLC data above and consistent with the hypothesis that production of the reutericyclins inhibits S. mutans growth in a dose-dependent manner. This is supported by the fact that purified MucF protein was able to complement the impaired growth phenotype of the Δ*mucF* strain on agar plates (8). On the other hand, spent media from a liquid culture of Δ*mucF* was not more inhibitory to further growth of S. mutans than spent media from a liquid culture of Δ*mucD*, and in fact more growth was observed in cultures using the spent Δ*mucD* media, when both spent media were mixed with an equal volume of fresh media (data not shown). However, as Δ*mucD* grows to a much higher density, it is likely that more nutrients in the Δ*mucD* spent media were depleted, and therefore the medium was not able to support growth to the same extent. It is also possible that the extracellular reutericyclin in the Δ*mucF* spent medium is not concentrated enough to have a significant effect on growth, (i.e., the growth inhibition in liquid culture is caused more by intracellular accumulation of reutericyclin rather than extracellular accumulation).

### Deletion of *mucG* reduces transcription of *mucD*, *mucE*, and *mucI*, while deletion of *mucH* reduces transcription of the entire *muc* operon.

To examine the role of the *mucG* and *mucH* genes that encode transcriptional regulators on the global transcriptome of B04Sm5, mRNA sequencing of mid-log-phase cultures of B04Sm5, Δ*mucG*, and Δ*mucH* was performed. Using DESeq2 differential abundance analysis, with a Benjamini-Hochberg corrected *P* value cutoff of 0.001, compared to the parent strain, B04Sm5, there were 106 genes with reduced mRNA expression and 218 genes with increased expression in Δ*mucG* only (Table S4). Meanwhile, compared to B04Sm5, there were 160 genes with reduced expression and 180 genes with increased expression in Δ*mucH* only (Table S5). There were also 269 genes differentially regulated compared to B04Sm5 in both Δ*mucG* and Δ*mucH*, with all but 11 of those genes changing expression the same direction in both mutants compared to B04Sm5 (Table S6). In terms of expression of the *muc* BGC specifically, in both cases, the deleted gene was the gene with the highest reduction in expression, as expected with deletion mutants (Table 1). The Δ*mucG* strain had modestly reduced (−1.5 to −2 log_2_-fold) expression of *mucD*, *mucE*, and *mucI*, while the Δ*mucH* strain had more significantly reduced (−2.1 to −8.5 log_2_-fold) expression of the entire operon (Table 1). Given the HPLC and growth phenotypes observed, it was surprising that Δ*mucG* had reduced transcription of *mucD*, *mucE*, and *mucI.* This suggested that the cause of the increased production of mutanocyclin and the reutericyclins observed in Δ*mucG* must occur at the proteomic or metabolomic level. To obtain an overview of the global transcriptomic effects of deletion of *mucG* or *mucH*, KOs from differentially regulated genes were projected onto a map of S. mutans metabolism using KEGG Mapper (https://www.genome.jp/kegg/mapper/color.html; Fig. S4). An interactive version of the map can be obtained by the reader by using Table S7 as input for the KEGG Mapper tool. Overall, the results were difficult to interpret, and few clear patterns were apparent. The large number of differentially expressed genes was also unexpected and is likely to reflect changes due to the presence of mutanocyclin and/or reutericyclins rather than direct regulation by MucG and MucH. Because Δ*mucG* and Δ*mucH* have opposing phenotypes, the large amount of overlap in the transcriptomes was surprising. It might be expected that Δ*mucH* may reflect the “least stressed” condition, based on the growth curve, and some trends in Δ*mucH* are similar to those seen when S. mutans is grown in “nonstressful” conditions (increased expression of *rmlA*); however, some trends do not (decreased expression of *accA* to *D*) (24).

**TABLE 1:** Differential expression of *muc* in the Δ*mucD*, Δ*mucF*, Δ*mucG*, and Δ*mucH* strains, compared to B04Sm5 (log_2_-fold change)

Name	Locus ID	Gene cluster ID	Gene callers ID	NCBI PGAP accession	NCBI PGAP annotation description	*ΔmucD*	*ΔmucF*	*ΔmucG*	*ΔmucH*
*mucA*	IBL27_RS09110	GC_00001982	1693			2.58564569	1.19925521		−7.1955871
*mucB*	IBL27_RS09105	GC_00001981	1692	WP_002277495.1	Thiolase family protein	1.32475972	−1.7582417		−4.7312389
*mucC*	IBL27_RS09100	GC_00001980	1691				−2.3188811		−6.4796598
*mucD*	IBL27_RS09095	GC_00001984	1690	WP_002308266.1	Nonribosomal peptide synthetase	−14.06934	−1.5771313	−1.8548904	−6.5515393
*mucE*	IBL27_RS09090	GC_00001986	1689	WP_002283498.1	Polyketide synthase	−3.5433655	−2.1506834	−2.0506685	−5.9316069
*mucF*	IBL27_RS09085	GC_00000014	1688	WP_002277499.1	HXXEE domain-containing protein	−2.7841312	−5.865363		−2.1839221
*mucG*	IBL27_RS09080	GC_00001987	1687	WP_002283497.1	TetR/AcrR family transcriptional regulator	−3.9166794	−3.1611157	−8.5142069	−3.5935604
*mucH*	IBL27_RS09075	GC_00001983	1686	WP_002277501.1	TetR/AcrR family transcriptional regulator	1.06763813			−10.557072
*mucI*	IBL27_RS09070	GC_00001979	1685	WP_002277502.1	DHA2 family efflux major facilitator superfamily (MFS) transporter permease subunit	−3.2679608	−3.2504807	−1.600364	−8.524352
*mucJ*	IBL27_RS09065				Small multidrug export protein	−2.5618472	−3.3842254		−2.6864964

Recently, the transcriptomic effects of addition of B04Sm5, Δ*mucD*, or Δ*mucF* to a complex *in vitro* oral microbial community were examined (10). By mapping the mRNA sequencing reads from the complex community in that study to the B04Sm5 genome, a comparison of the transcriptome of Δ*mucD* and Δ*mucF* to Δ*mucG* and Δ*mucH* was possible; however, it is crucial to note that the context of the Δ*mucD* and Δ*mucF* transcriptomes is a community, whereas the Δ*mucG* and Δ*mucH* transcriptomes are single-species cultures (Table 1; Table S8 and S9). Interestingly, although Δ*mucF* and Δ*mucG*, had similar phenotypes and Δ*mucD* and Δ*mucH* had similar phenotypes, the of trends their transcriptomes was small. Altogether, these transcriptomic data indicate that MucH is an activator of transcription of the *muc* BGC, while the role of MucG is less clear.

### Δ*mucG* is significantly better than B04Sm5 at preventing the growth of commensal oral streptococci, while Δ*mucH* is worse, and Δ*mucI* inhibits only Streptococcus gordonii.

**FIG 3:**
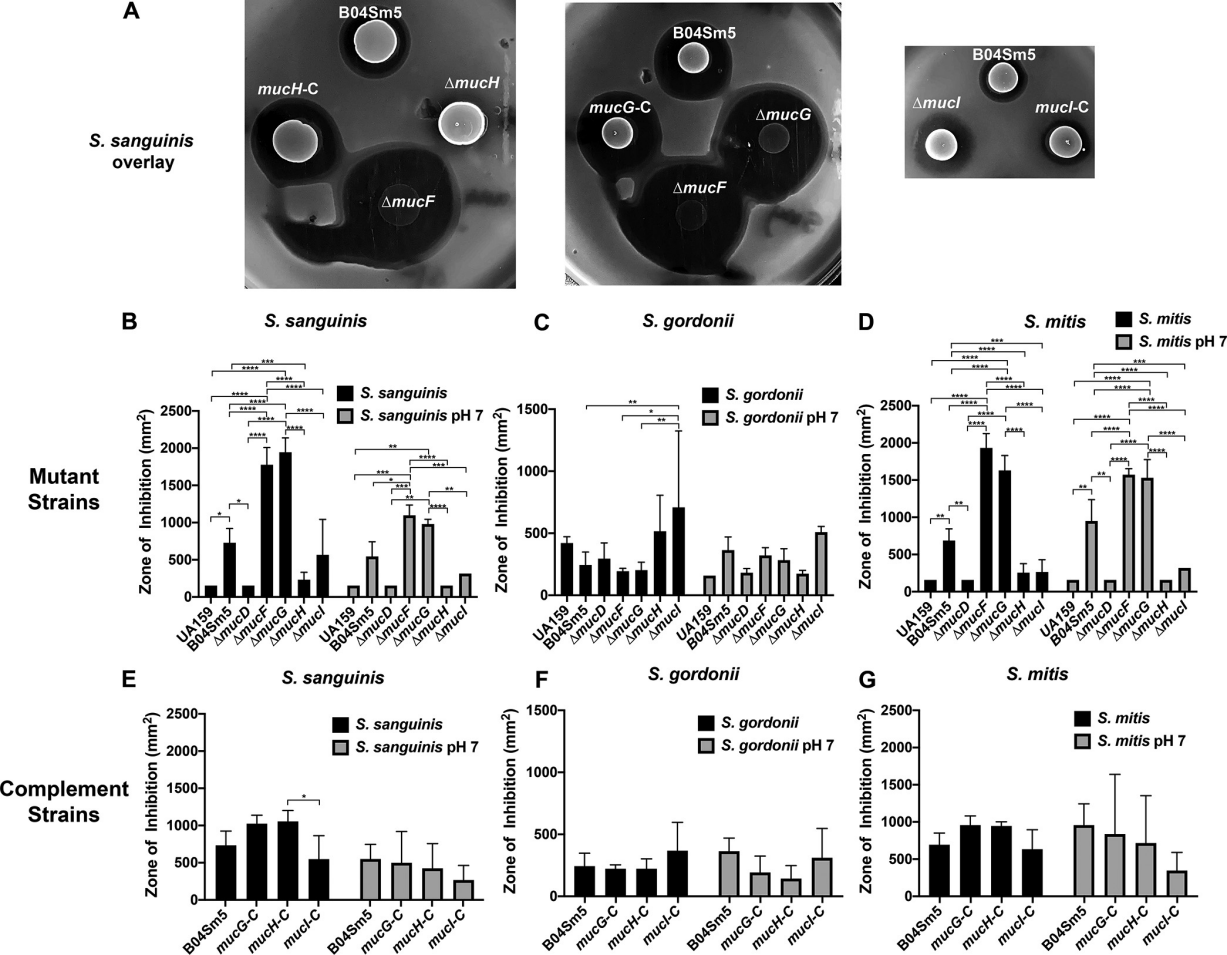
Loss of *mucG*, *mucH*, or *mucI* affects the ability of B04Sm5 to inhibit the competing, health-associated bacteria S. sanguinis, S. gordonii, and S. mitis. Deferred antagonism assay was performed as described under Materials and Methods. Cultures of the indicated S. mutans strains were spotted on to BHI agar and incubated overnight. Cultures of the indicated competing organism were added to BHI soft agar and used to overlay the plates containing the S. mutans. (A) Three replicate assays were performed, and representatives of the brain heart infusion (BHI) plates with an S. sanguinis overlay are shown. (B to G) Bar graphs representing quantification of the zones of inhibition observed with the indicated S. mutans strain and competing species. Error bars represent the standard deviation, and asterisks denote statistical significance between indicated pairs as determined by a Tukey’s multiple-comparison test following a two-way way analysis of variance (ANOVA). *, *P* < 0.05; **, *P* < 0.01; ***, *P* < 0.001; ****, *P* < 0.0001.

To examine the ability of the Δ*mucG*, Δ*mucH*, and Δ*mucI* strains to inhibit the growth of neighboring commensal organisms, colonies of B04Sm5, UA159, the *muc* deletion mutants, and complement strains were spotted on BHI agar or BHI agar buffered to pH 7. Following 24 h of growth, these plates were overlaid with soft agar containing either S. gordonii, Streptococcus sanguinis, or Streptococcus mitis, which are all oral streptococci that are generally health-associated and inversely correlated with S. mutans and with dental caries (2, 3). Similar to what was observed in the growth curves shown in Fig. 2, the phenotype of Δ*mucG* mirrored that of Δ*mucF*, while the phenotype of Δ*mucH* mirrored that of Δ*mucD* (Fig. 3). Δ*mucF* and Δ*mucG* had significantly larger zones of inhibition against S. sanguinis and S. mitis compared to the parent strain, B04Sm5. Meanwhile, Δ*mucD* and Δ*mucH* had significantly smaller zones of inhibition against S. sanguinis and S. mitis compared to B04Sm5 (Fig. 3). Buffering the BHI agar to pH 7 reduced the zones of inhibition in most cases, but these phenotypic differences were still observed (Fig. 3). This effect was not observed against S. gordonii, consistent with the less dramatic effect of *muc* against S. gordonii observed previously (8). Interestingly, Δ*mucI* displayed a similar or slightly reduced zone of inhibition against *S. sangunis* and S. mitis but had a significantly increased zone of inhibition against S. gordonii on unbuffered BHI agar, but not on BHI agar buffered to pH 7 (Fig. 3). As seen in the previous assays, complementation of the Δ*mucG*, Δ*mucH*, or Δ*mucI* mutants faithfully restored the phenotype of the parent strain (Fig. 3).

## DISCUSSION

Dental caries is caused by a dysbiotic dental plaque microbiome that creates an acidic microenvironment adjacent to the tooth surface, which demineralizes the protective enamel, leading to infection of the dentin and irreparable damage if the process is unchecked (1, 2). S. mutans contributes to the formation of this pathogenic community through its exceptional ability to form biofilms in the presence of sucrose, generate acid from many types of dietary carbohydrates, and tolerate an acidic environment (25). Along with indirectly inhibiting health-associated neighbors (which are generally much less acid-tolerant than S. mutans) through acid production, S. mutans can also directly kill and inhibit the growth of neighboring bacteria through production of small molecules such as class 1 bacteriocins (e.g., mutacin lanthibiotic), nonribosomal peptides (NRPs), polyketides (PKs), and hybrids of these (e.g., reutericyclin) (8, 25, 26). A large amount of diversity within the S. mutans species has been well documented, in terms of both genomic content and phenotypes related to virulence traits as mentioned above (27–31). However, it has been difficult to directly link carriages of particular genotypes to dental caries incidence (25, 32, 33).

The updated pangenome provided by this study concurs with previous S. mutans pangenomic analyses, indicating that S. mutans has approximately 1,400 core genes and well over 3,000 genes in total (29, 31). Of the 244 S. mutans genomes available via the RefSeq database (as of June 2021), a subset of 35 genomes encode the *muc* BGC. *muc* is homologous to the reutericyclin BGC (Fig. S1), which was first elucidated in L. reuteri, and produces reutericyclin, an acylated tetramic acid that has potent antimicrobial activity against a broad range of Gram-positive organisms (15). All 35 of the S. mutans *muc* BGCs encode 10 genes: *mucABC*, which currently have unknown function (but are predicted to be tailoring enzymes and are homologous to diacetylphloroglucinol biosynthesis machinery from Pseudomonas fluorescens), the MucD NRPS, the MucE PKS, the MucF acylase (which cleaves the acyl chain from reutericyclin to yield mutanocyclin), the TetR/AcrR family transcriptional regulators MucG and MucH, the MucI DHA2 family transporter, and MucJ, which is predicted to be a small multidrug export protein. Due to differing versions of antiSMASH software used, MucJ was identified by Hao et al. (9) but not by Liu et al. (7) or Tang et al. (8). Two recent studies independently elucidated that the major product of S. mutans *muc* is mutanocyclin (8, 9), which has the same core tetramic acid structure as reutericyclin but is lacking the acyl chain. One of these studies also detected production of several types of reutericyclin molecules (differing on the length and saturation of the acyl chain), in addition to mutanocyclin production (8), while the other study did not (9). Although differences in growth conditions and/or biochemical detection methods may account for this, it is also possible that, given the sequence divergence of the *muc* cluster between the strains used, S. mutans B04Sm5 produces reutericyclin and S. mutans 35 does not (although *mucF*, specifically, is identical between the two strains). Mutanocyclin was anti-inflammatory in a murine model (9) and had limited antimicrobial activity, selectively inhibiting the growth of L. fermentum in a complex *in vitro* oral microbiome (10).

The origin of *muc* remains unclear. In the L. reuteri reutericyclin BGC, it was hypothesized that the homologs to *mucABC* and *mucDE* had distinct phylogenetic origins (15). This was supported by the fact that *rtcN* (*mucD* homolog) and *rtcA* (*mucA* homolog) appeared to each originate from different species outside S. mutans and L. reuteri (15). The later study by Hao et al. (9) also identified *muc* in Sarcina troglodytae and Streptococcus macacae, which are found in the oral cavities of nonhuman primates (9). Due to modest (40 to 60% homology) between the *muc* and *rtc* BGCs, direct horizontal gene transfer between S. mutans and L. reuteri was hypothesized to be unlikely (15). Within S. mutans, however, there is little variability in *muc*, with the cluster being 99.4% identical at the protein level, with only 23 sites across *mucABCDEFGHI* (3,832 amino acids in total) differing among the 35 strains encoding the gene cluster (https://github.com/jonbakerlab/Smutans-pangenome). Despite these interstrain differences in *muc* being small, the phylogenetic analysis of *muc* performed here indicated three main lineages of the cluster in S. mutans (Fig. 1C). These lineages did not line up with overall S. mutans phylogeny, implying horizontal gene transfer of the cluster within S. mutans (Fig. 1D). Overall, the data suggest that there may have been one ancestral *muc* within the clade of S. mutans containing the most frequent carriage of *muc*, and that this cluster spread to other more distant S. mutans clades via horizontal gene transfer. The observations that *muc* is absent in many of the strains within the clade containing the likely ancestral gene cluster also suggest that loss of *muc* is common, and selection pressure may be against retaining *muc* under many conditions. Co-occurrence analysis using Coinfinder (14) identified 46 genes in the S. mutans pangenome that co-occurred with *muc* more than would be expected based on phylogeny alone. This indicates that either carriage of *muc* with these genes may provide an advantage compared to carriage of *muc* or the other genes alone. Many of the co-occurring genes, including the type A2 lanthipeptide (GC00001893) and the other hybrid NRPS/PKS (GC00001895), are more broadly distributed phylogenetically than *muc* (Fig. 1D). Coinfinder also failed to find any genes that contraindicated carriage of *muc* (i.e., by increasing sensitivity to reutericyclin, for example); however, since *muc* is found in only 14% of S. mutans genomes, and is limited to several clades, it is possible that these types of interactions exist in S. mutans, and *muc* has simply not sampled enough genetic backgrounds to make these genes apparent statistically. Further examination of the genes and BGCs that correlate with *muc* will determine whether these co-occurring genes increase resistance to reutericyclin or provide synergy with *muc* through another mechanism.

This study also examined the role of the genes encoding the MucG and MucH TetR/AcrR family transcriptional regulators and the MucI DHA2 family transporter. The L. reuteri *rtc* BGC also encodes two TetR/AcrR transcriptional regulators (*rtcRS*) and a transporter (*rtcT*) with varied homology to their counterparts in S. mutans (∼20% for *rtcRS/mucGH* and 64% for *rtcT/mucI*) (15). These three genes conferred reutericyclin resistance in *L. reuteri*, based on the fact that deletions of *rtcT* and *rtcRS* in reutericyclin-producing strains were lethal, while deletions of these genes in mutants that did not produce reutericyclin were possible (15). Therefore, RtcT was predicted to export reutericyclin from the cells, and RtcR/RtcS were predicted to be activators of *rtcT* transcription (15). Deletions of the cognate homologous genes, *mucG*, *mucH*, and *mucI* were accomplished in S. mutans B04Sm5, likely, at least in part, because most of the reutericyclin is cleaved into the much less cytotoxic mutanocyclin by *mucF* (8). Future research attempting to delete *mucG*, *mucH*, or *mucI* in conjunction with *mucF* will test this hypothesis.

Deletion of *mucG* or *mucH* caused opposing phenotypes. Δ*mucG* had increased production of mutanocyclin and reutericyclin, reduced growth, and a larger zone of inhibition of against S. sanguinis and S. mitis, reminiscent of the phenotype of the Δ*mucF* strain, which also produces more reutericyclin (but does not make mutanocyclin, unlike Δ*mucG*) (Fig. 2 and 3) (8). Meanwhile, Δ*mucH* mimicked the phenotype of Δ*mucD*, with reduced mutanocyclin and reutericyclin production, enhanced growth, and a significantly reduced zone of inhibition against S. sanguinis and S. gordonii (Fig. 2 and 3) (8). Δ*mucD* does not produce either reutericyclin or mutanocyclin (8). The simple hypothesis from these data would be that *mucG* is a repressor of *muc* and that *mucH* is a transcriptional activator. Based on the transcriptomics, this appears to be the case for *mucH*, as the entire *muc* BGC experienced significantly reduced expression upon its deletion. The role of *mucG* is not as clear, however, as transcription of *muc* was not increased in Δ*mucG*, and in fact expression of *mucD*, *mucE*, and *mucI* was reduced (however, to a lesser extent than was observed in Δ*mucH*). Furthermore, transcription of *mucF* was not decreased, so the ratio of MucD to MucF in Δ*mucG* would appear to be less in favor of producing reutericyclin, which is contrary to the observed phenotype. Since TetR/AcrR regulators typically bind cofactors that affect their transcriptional regulatory activity (34), it is possible that cofactors affect activity of *mucG* and *mucH* and may explain the discrepancy between phenotype and expression level observed in Δ*mucG.* For example, *mucG* may bind either reutericyclin or mutanocyclin and adjust transcription of *muc* to limit reutericyclin production. Alternatively, reutericyclin and mutanocyclin production may be affected in Δ*mucG* by changes at the proteomic or metabolomic level. Additional studies are needed to further elucidate regulation of *muc* by *mucG* and *mucH* and identify the cofactors involved.

The role of MucI remains unclear. The reduced amount of mutanocyclin and reutericlins in the supernatant of Δ*mucI* would seem to indicate that MucI exports one or both of these molecules, which is in line with the hypothesis that the *mucI* homolog, *rtcT*, exports reutericyclin in L. reuteri. However, deletion of *mucI* also caused enhanced growth, as was seen in Δ*mucD* and Δ*mucH* (Fig. 2), which is the opposite of what was observed upon deletion of *rtcT* in L. reuteri (15). Further research is needed to determine the function of the *mucI* transporter.

Overall, this study provides oral microbiology researchers with an updated phylogenetic analysis of S. mutans and also an updated and in-depth pangenome analysis that can be used to examine carriage of various genes and metabolic functions across S. mutans phylogeny. This study also shows that *mucG*, *mucH*, and *mucI* all affect production of mutanocyclin and reutericyclins by the *muc* BGC and therefore affect S. mutans overall physiology. Research in progress will further elucidate the regulation and expression of the *muc* BGC and the production and roles of reutericyclin and mutanocyclin in the ecology and virulence potential of S. mutans.

## MATERIALS AND METHODS

### Bacterial strains and growth conditions.

All of the strains used in this study are listed in Table 2. The S. mutans strains UA159 (35), B04Sm5 (33), Δ*mucD* (8), and Δ*mucF* (8) have been described previously. S. mutans was maintained on brain heart infusion (BHI) agar plates (BD/Difco, Franklin Lakes, NJ) at 37°C in a 5% (vol/vol) CO_2_/95% air environment. Where applicable, antibiotics were added to a final concentration of 5 μg/mL for erythromycin and 1 mg/mL for spectinomycin.

**TABLE 2:** Strains, plasmids, and primers used in this study

Strain, plasmid, or primer	Description (reference[s] or source, if not this study)
Strains	
Streptococcus mutans	
* *UA159	Genomic type strain (does not encode *mucA* to *mucJ*) (35)
* *B04Sm5	Parent strain (encodes *mucA* to *mucJ*) (32, 33)
* *Δ*mucD*	*mucD* deletion strain (8)
* *Δ*mucF*	*mucF* deletion strain (8)
* *Δ*mucG*	*mucG* deletion strain
* *Δ*mucH*	*mucH* deletion strain
* *Δ*mucI*	*mucI* deletion strain
* mucG-C*	*mucG* complement strain
* mucH*-C	*mucH* complement strain
* mucI*-C	*mucI* complement strain
Streptococcus gordonii ATCC 10558	Used in deferred antagonism assay (ATCC)
Streptococcus sangunis SK36	Used in deferred antagonism assay (ATCC)
Streptococcus mitis F0392	Used in deferred antagonism assay (ATCC)
Escherichia coli NEB 5α	Host for the plasmids constructed in this study (New England Biolabs)
Plasmids	
* *pCAPB2	Construct containing *specR* gene
* *pUC19	Vector backbone to make deletion constructs
* *pΔmucG	Construct to make the Δ*mucG* strain
* *pΔmucH	Construct to make the Δ*mucH* strain
* *pΔmucI	Construct to make the Δ*mucI* strain
* *pBGE	Vector backbone containing erythromucin resistance cassette (gift from José Lemos)
* *pmucG-C	Construct to make the *mucG*-C strain
* *pmucH-C	Construct to make the *mucH*-C strain
* *pmucI-C	Construct to make the *mucI*-C strain
Primers	
* *spec_fwd	Used to amplify *specR,* CAATTTTTTTATAATTTTTTTAATCTGTTA
* *spec_rev	Used to amplify *specR,* ATAACTAATAACGTAACGTG
* *mucG_KO_L-fwd	Used to make the *mucG*-C strain, CATGATTACGCCAAGCTTGCATGCCTGCAGGCCTTTAAATAAGTTTAACAGCC
* *mucG_KO_L-rev	Used to make the *mucG*-C strain, ATAACAGATTAAAAAAATTATAAAAAAATTGTACTTTCCTCCTGCTTATAAATG
* *mucG_KO_R-fwd	Used to make the *mucG*-C strain, CTCTTGCCAGTCACGTTACGTTATTAGTTATCGATAGAACTGGGATTGCTTC
* *mucG_KO_R-rev	Used to make the *mucG*-C strain, TCACGACGTTGTAAAACGACGGCCAGTGAATTCTAAAACAAGACAGACACACAGC
* *mucH_KO_L-fwd	Used to make the *mucH*-C strain, CATGATTACGCCAAGCTTGCATGCCTGCAGCATTTATAAGCAGGAGGAAAGTA
* *mucH_KO_L-rev	Used to make the *mucH*-C strain, ATAACAGATTAAAAAAATTATAAAAAAATTGGAAGCAATCCCAGTTCTATCG
* *mucH_KO_R-fwd	Used to make the *mucH*-C strain, CTCTTGCCAGTCACGTTACGTTATTAGTTATCTGTGTGTCTGTCTTGTTTTAC
* *mucH_KO_R-rev	Used to make the *mucH*-C strain, TCACGACGTTGTAAAACGACGGCCAGTGAATTCAGTTAAAATGGCACCGCCTAG
* *mucI_KO_L-fwd	Used to make the *mucI*-C strain, ACCATGATTACGCCAAGCTTGCATGCCTGCAGAAAACGACAGATTCTAATGTG
* *mucI_KO_L-rev	Used to make the *mucI*-C strain, AAATAACAGATTAAAAAAATTATAAAAAAATTGAAATGTAGAAACCATAACCAG
* *mucI_KO_R-fwd	Used to make the *mucI*-C strain, ATCTCTTGCCAGTCACGTTACGTTATTAGTTATTTTTAACCCTCTTTCATTATTAT
* *mucI_KO_R-rev	Used to make the *mucI*-C strain, AGTCACGACGTTGTAAAACGACGGCCAGTGAATTCTCTAGCTGAAAATCATTTTC
* *pmucG-C erm F	Used to make the pmucG-C, GAGCAAGGTTTCGACTCTAGTTTTAACCCTCTTTCATTATTATTTTAAATC
* *pmucG-C erm R	Used to make the pmucG-C, TGTTTTACAACCGGGTGTACTTATATAAAAATTTTATAAGAGGATAAGCTTTTG
* *pmucH-C erm F	Used to make the pmucH-C, GAGCAAGGTTTCGACTCTAGTAAGATGTCTTCCTTTCTTATTAAAATATG
* *pmucH-C erm R	Used to make the pmucH-C, TGTTTTACAACCGGGTGTACTTATTTCCAAAAGATATCTTCAATTTTCA
* *pmucI-C erm F	Used to make the pmucI-C, GAGCAAGGTTTCGACTCTAGTTTTCCCTTAATTTAACAACTTGTT
* *pmucI-C erm R	Used to make the pmucI-C, TGTTTTACAACCGGGTGTACCTAAATAGCTTGCTTTTCGATTG

### Pangenome and phylogenetic analyses.

Pangenome analysis was performed using the anvi'o pangenomics Snakemake workflow on 244 S. mutans strains from NCBI, listed in Table S1, using DIAMOND, a Minbit parameter of 0.5, and an MCL inflation of 9.0 (13, 36). Phylogenomics was performed using anvi'o (13, 36). The pangenome was used to identify an optimal set of core genes to perform concatenated protein sequence phylogeny on, as described in the tutorial for the anvi'o pangenomics tutorial (https://merenlab.org/2016/11/08/pangenomics-v2/). The pangenomics analysis identified 348 single-copy genes encoded by all 244 genomes (i.e., single-copy core genes [SCGs]). Filtering by a minimum geometric homogeneity index of 1 left 270 core genes. Low functional homogeneity indices of these genes result in only 1 or 2 genes passing all filters; therefore, the maximum functional homogeneity index was increased to 0.9925, leaving 12 optimal core genes on which to base the phylogenetic analysis of the 244 genomes (plus Streptococcus sobrinus as an outgroup). A list of the gene clusters in each genome and the phylogenetic tree based on specific core genes were used as input for Coinfinder (14) to identify genes correlated with *muc* using the Bonferroni correction option. The output of Coinfinder was used by Cytoscape (37) to draw the correlation network in Fig. S2.

### Generation of recombinant strains.

**(i) *mucG*, *mucH*, and *mucI* strains.** B04Sm5 derivatives were constructed with the *mucG*, *mucH*, or *mucI* genes replaced by a spectinomycin resistance gene (specR), as previously described (8). Briefly, a 1,010-bp fragment containing the spectinomycin resistance gene (specR) was amplified from pCAPB2 with primers spec_fwd and spec_rev. The left and right (∼500 bp each) flanking regions of *mucG*, *mucH*, or *mucI* were amplified from the genomic DNA of S. mutans B04Sm5 with the primer pairs of mucD_KO_L-fwd/mucD_KO_L-rev, mucD_KO_R-fwd/mucD_KO_R-rev, mucG_KO_L-fwd/mucG_KO_L-rev, mucG_KO_R-fwd/mucG_KO_R-rev, mucI_KO_L-fwd/mucI_KO_L-rev, and mucI_KO_R-fwd/mucI_KO_R-rev, respectively. These three PCR products were assembled with a double digested pUC19 (PstI and EcoRI) using a NEBuilder HiFi DNA assembly kit (New England Biolabs, USA), which resulted in the vectors pΔmucG, pΔmucH, and pΔmucI, respectively. These ligation products were transformed into E. coli NEB 5α cells, and positive clones were selected on LB agar medium containing 100 μg/mL spectinomycin. Vector clones were designated pΔmucG, pΔmucH, and pΔmucI and verified by restriction analysis and sequencing. The disruption cassettes were amplified from constructs using primer pairs muc/G_KO_L-fwd/mucH_KO_R-rev, mucH_KO_L-fwd/mucG_KO_R-rev, and mucI_KO_L-fwd/mucI_KO_R-rev, respectively. PCR products were digested by DpnI and then purified using the QIAquick PCR purification kit (Qiagen, USA). The disruption cassettes were transformed to S. mutans B04Sm5 by a previously reported protocol (38). Δ*mucG*, *ΔmucH*, and Δ*mucI* deletion mutants were selected by growth on BHI agar supplemented with 1 mg/mL spectinomycin and confirmed by PCR and sequencing.

**(ii) *mucG*-C, *mucH*-C, and *mucI-C* complement strains.** Complement strains of Δ*mucG*, *ΔmucH*, and Δ*mucI* were generated using a previously described protocol (39). Briefly, single-copy genomic insertion of either *mucG*, *mucH*, or *mucI* into the *gtfA* locus of the cognate Δ*mucG*, Δ*mucH*, or Δ*mucI* strain was performed. Primers pmtaG-C erm F and pmtaG-C erm R were used to amplify the *mucG* open reading frame, as well as the 129-bp intergenic region upstream (between *mucG* and *mucF*) of *mucG.* Primers pmtaH-C erm F and pmtaH-C erm R were used to amplify the *mucH* open reading frame, as well as the 178-bp intergenic region upstream of *mucH* (between *mucH* and *mucI*). Primers pmtaI-C erm F and pmtaI-C erm R were used to amplify the *mucI* open reading frame, as well as the 178-bp intergenic region upstream of *mucI* (between *mucH* and *mucI*). The streptococcal integration vector pBGE was a gift from Jose Lemos and has been previously described (39). pBGE was linearized using XbaI and BsrG1, and the *mucG*, *mucH*, and *mucI* PCR products were ligated into pBGE using the Gibson assembly cloning kit (New England Biolabs) to generate pmucG-C, pmucH-C, and pmucI-C, respectively. These ligation products were transformed into E. coli NEB 5α cells, and positive clones were selected on LB agar medium containing 500 μg/mL erythromycin. The integrity of each construct was confirmed by sequencing. pmucG-C, pmucH-C, and pmucI-C were transformed into Δ*mucG* Δ*mucH*, and Δ*mucI* to generate the strains *mucG-C*, *mucH-C*, and *mucI*-C, respectively. Selection was performed on BHI agar containing 5 μg/mL erythromycin, and the integrity of both the deletion and complementation loci was confirmed by sequencing.

### HPLC.

50-mL aliquots of BHI medium containing 1% glucose were inoculated with a loop of glycerol stock of B04Sm5, Δ*mucD*, Δ*mucF*, Δ*mucG*, Δ*mucH*, Δ*mucI*, *mucG-*C, *mucH*-C, or *mucI*-C and incubated at 37°C under 5% CO_2_/95% air. After 16 h, 1 g of Amberlite XAD7-HP resin (Sigma-Aldrich, Burlington, MA, USA) was added to the cultures. The cultures were then incubated for an additional 36 h, after which the resin was recovered using a coffee filter. The resin was washed twice with 10 mL of molecular grade water and extracted with 5 mL of ethyl acetate. The organic phase was decanted and evaporated, with the resulting pellet resuspended in 100 μL of methanol. Extracts were monitored at 280 nm during separation using an Agilent Technologies (Santa Clara, CA, USA) 1200 series HPLC equipped with a Kinetex C18 100 Å LC column (5 μm, 150 × 2.1 mm) (Phenomenex, Inc., Torrance, CA, USA) as follows: Solvent A contained HPLC-grade H_2_O and trifluoroacetic acid (TFA) (999:1, vol/vol), whereas solvent B contained HPLC-grade acetonitrile (CH_3_CN) and trifluoroacetic acid (TFA) (999:1, vol/vol). 0 to 15 min, 30% solvent B; 15 to 16 min, ramp 30 to 100% solvent B; 16 to 25 min, 100% solvent B; 26 to 27 min, ramp 100 to 30% solvent B; and 28 to 35 min, 30% solvent B.

### Growth curve.

Overnight cultures of UA159, B04Sm5, Δ*mucD*, Δ*mucF*, Δ*mucG*, Δ*mucH*, *ΔmucI*, *mucG*-C, *mucH*-C, or *mucI*-C were normalized to the optical density at 600 nm (OD_600_) of the lowest-density culture (Δ*mucF*) with BHI. A total of 10 μL of these cultures were added to 200 μL of BHI in a 96-well plate. Growth was monitored using a Tecan Infinite Nano. OD_600_ was measured every hour for 20 h under 37°C, with 5 s of shaking prior to each reading. Eight replicates of each strain were monitored.

### Deferred antagonism assay.

The deferred antagonism assay was performed as previously described (8). Briefly, 8 μL of overnight cultures of UA159, B04Sm5 Δ*mucD*, Δ*mucF*, Δ*mucG*, Δ*mucH*, Δ*mucI*, *mucG*-C, *mucH*-C, or *mucI*-C was spotted onto BHI + 1% agar or BHI + 1% agar that was buffered to pH 7 with 1 M KH_2_PO_4_/K_2_HPO_4_, pH 7.5, and incubated overnight at 37°C under 5% CO_2_/95% air. The following day, the plates were sterilized using the sterilization setting (90 s) in a GS Gene Linker UV Chamber (Bio-Rad, Inc.). 500 μL of overnight cultures of S. sanguinis SK36, S. gordonii ATCC 10558, or S. mitis F0392 was added to 5 mL molten BHI + 0.75% agar that had been cooled to 40°C, and this was used to overlay the plates with the S. mutans colonies. The agar overlay was allowed to solidify at room temperature, and then the plates were incubated overnight at 37°C under 5%CO_2_/95% air. Zones of inhibition were measured the following day using a ruler.

### RNA sequencing.

B04Sm5, Δ*mucG*, and Δ*mucH* were grown in BHI at 37°C in 5% CO_2/_95% air to mid-log-phase (OD_600_ of ∼0.65 for B04Sm5 and Δ*mucH* and 0.25 for Δ*mucG*, because of the growth defect phenotype). 1-mL aliquots of B04Sm5 and Δ*mucH* and 50-mL aliquots of Δ*mucG* were pelleted and frozen at −80°C. Total RNA was extracted using a RNeasy power microbiome kit (Qiagen, Inc.) according to the manufacturer’s instructions. PolyA tails were then added to the rRNA-depleted RNA using E. coli poly(A) polymerase (New England Biolabs). rRNA was depleted using a RiboMinus Bacteria rRNA depletion kit (Thermo Fisher Scientific) according to the manufacturer’s instructions. RNA was checked for quality at each step using a Qubit (Thermo Fisher Scientific) and a TapeStation (Agilent Technologies). RNA libraries were then constructed using the PCR-cDNA sequencing kit (Oxford Nanopore Technologies) and sequenced on a GridION using a R9.4.1 flow cell (Oxford Nanopore Technologies). Basecalling was performed using Guppy version 4.0.11/MinKNOW version 20.06.09. Reads were mapped to the B04Sm5 genome using minimap2. The number of reads which mapped to each gene was determined using featureCounts (40). Differential abundance between strains was determined using DeSeq2 (41) implemented using R (www.r-project.org). Genes with KEGG annotations were mapped on to the metabolic network using the KEGG color pathway tool (https://www.genome.jp/kegg/mapper/color.html). Minimap2 was used to map reads from the B04Sm5-, Δ*mucD-*, and Δ*mucF-*amended microbial communities previously published (10) (available at NCBI, PRJNA773113) to the genome of B04Sm5. As above, the number of reads mapped to each gene was determined using featureCounts. Differential abundance between strains was determined using DeSeq2.

### Data availability.

The raw mRNA sequencing reads used in this study have been deposited in the Sequence Read Archive (SRA) database, and the BioProject accession number is PRJNA801007. The full S. mutans pangenome of 244 strains is available at https://github.com/jonbakerlab/Smutans-pangenome.
