# Supplementary material for: mucG, mucH, and mucI Modulate Production of Mutanocyclin and Reutericyclins in Streptococcus mutans B04Sm5
Source: J Bacteriol. 2022 Apr 11;204(5):e00042-22. doi: 10.1128/jb.00042-22 (PMC9112991; doi:10.1128/jb.00042-22)
Supplement: Supplemental file 7 — Figures S1-S4. Download jb.00042-22-s0007.pdf, PDF file, 3.1 MB [file jb.00042-22-s0007.pdf]

***mucG, mucH,* and *mucI* modulate production of mutanocyclin and reutericyclins in *Streptococcus mutans* B04Sm5**

**Supplemental material: Supplmental figures and figure legends.**

**Figure S1: Organization of the *muc* BGC, and homology to the *rtc* BGC in *L. reuteri*.** Diagram illustrating organization of the *muc* and *rtc* BGCs, the predicted functions of each gene product, and homology between cognate genes. Low homology between *mucF* and *rtcP,* and between the transcriptional regulators is indicated by red text and dashed lines. Adapted from Reference (8).

**Figure S1**

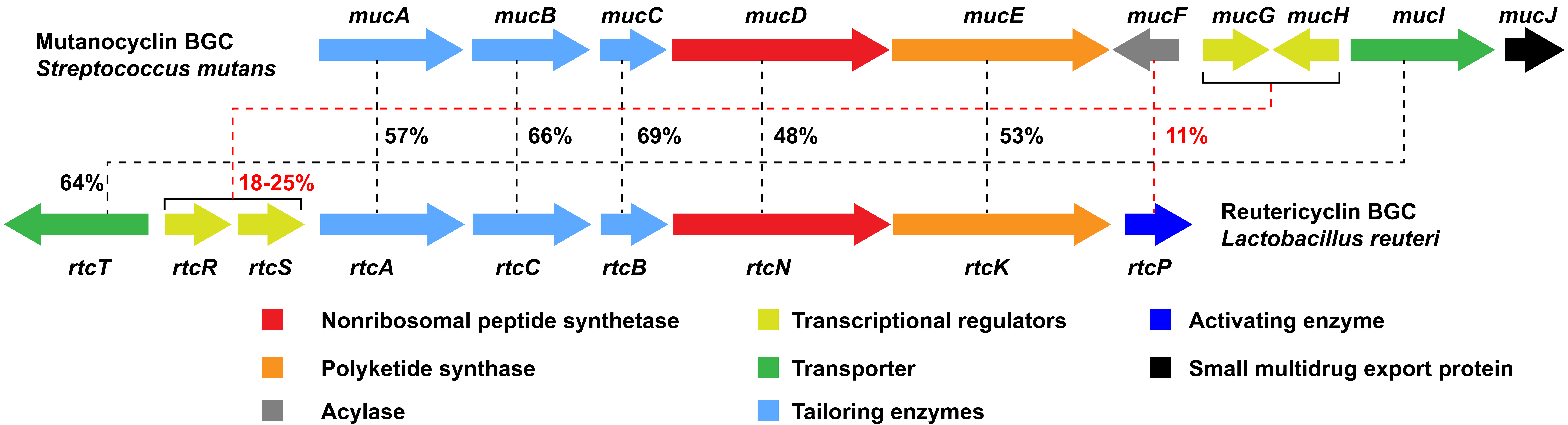
Mutanocyclin BGC
*Streptococcus mutans*
*mucA*
*mucB*
*mucC*
*mucD*
*mucE*
*mucF*
*mucG*
*mucH*
*mucI*
*mucJ*
57%
66%
69%
48%
53%
11%
64%
18-25%
*rtcT*
*rtcR*
*rtcS*
*rtcA*
*rtcC*
*rtcB*
*rtcN*
*rtcK*
*rtcP*
Reutericyclin BGC
*Lactobacillus reuteri*
Nonribosomal peptide synthetase
Polyketide synthase
Acylase
Transcriptional regulators
Transporter
Tailoring enzymes
Activating enzyme
Small multidrug export protein

**Figure S2: *muc* pangenome correlation network.** Correlation network illustrating the *muc* genes (yellow diamonds) and genes they are correlated with, based on Coinfinder analysis. Nodes are other genes. Node color indicates presence of *muc* correlated genes in the shell (gray) or cloud (blue) pangenome. Node shape indicates gene clusters of interest: diamond = *muc*; chevron = hybrid NRPS/PKS; triangle = type-A2 lanthipepetide; circle = all other genes. Edges represent positive correlations only, and edge thickness indicates the p-value of the correlation.

# Figure S2

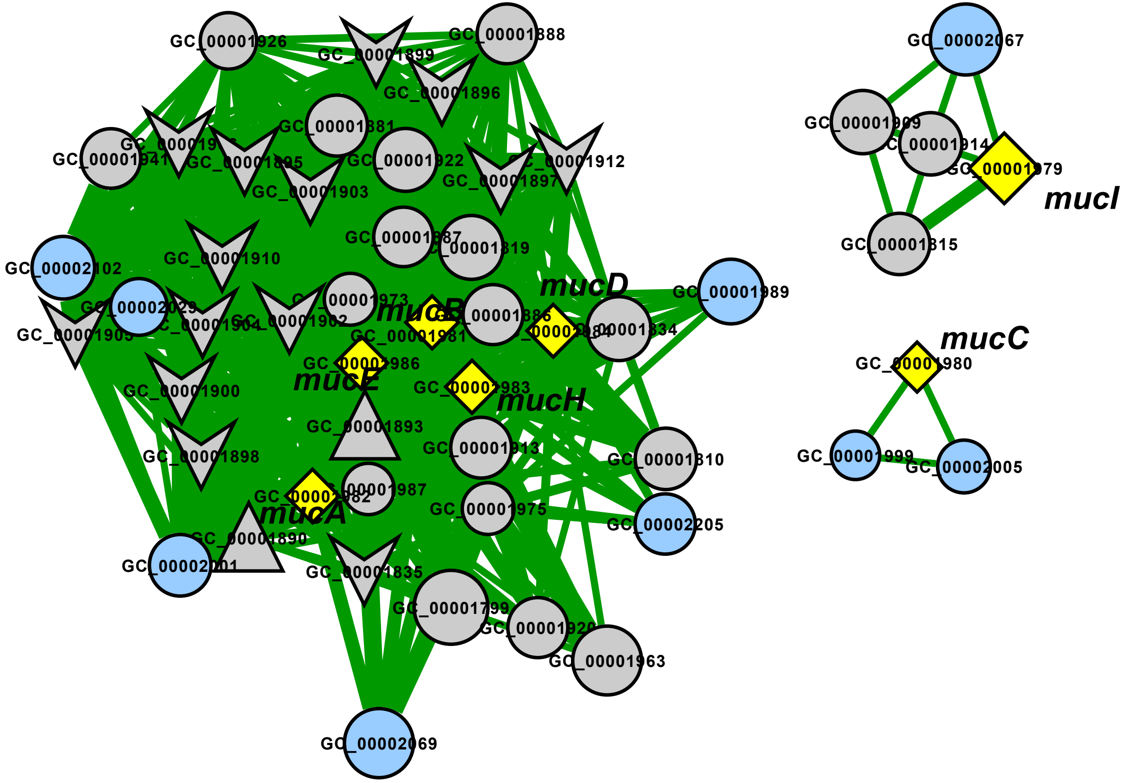
GC_00001926
GC_00001888
GC_00001899
GC_00001896
GC_00001881
GC_00001941
GC_00001895
GC_00001922
GC_00001912
GC_00001897
GC_00001903
GC_00001887
GC_00001910
GC_00002102
mucD
GC_00001989
mucB
GC_00001886
GC_00002029
GC_00001904
GC_00001902
GC_00001981
GC_00001834
GC_00001905
GC_00001986
mucE
GC_00001983
mucH
GC_00001900
GC_00001893
GC_00001913
GC_00001810
GC_00001898
GC_00001987
GC_00001982
mucA
GC_00001975
GC_00002205
GC_00001890
GC_00002001
GC_00001835
GC_00001799
GC_00001920
GC_00001963
GC_00002069
GC_00002067
GC_00001909
GC_00001914
GC_00001979
mucI
GC_00001815
mucC
GC_00001980
GC_00001999
GC_00002005

**Figure S3: *S. mutans* phylogeny.** Phylogenetic tree of 244 *S. mutans* genomes based on the concatentated protein sequences of 12 core genes, as described in the Materials and Methods. The tree is annotated with 3 layers indicating the presence of *muc,* GC0000189 (type-A2 lanthipeptide which was correlated with muc in Figure S2), and GC00001910 (hybrid NRPS/PKS which was correlated with *muc* in Figure S2). Presence/absence bars in the *muc* layer are colored based on the position of the *muc* BGC of the cognate genome in the *muc* phylogenetic tree in Figure 1C. *S. mutans* genomes of interest are labeled (B04Sm5, UA159, UA140, NN20205, and 35).

**Figure S3**

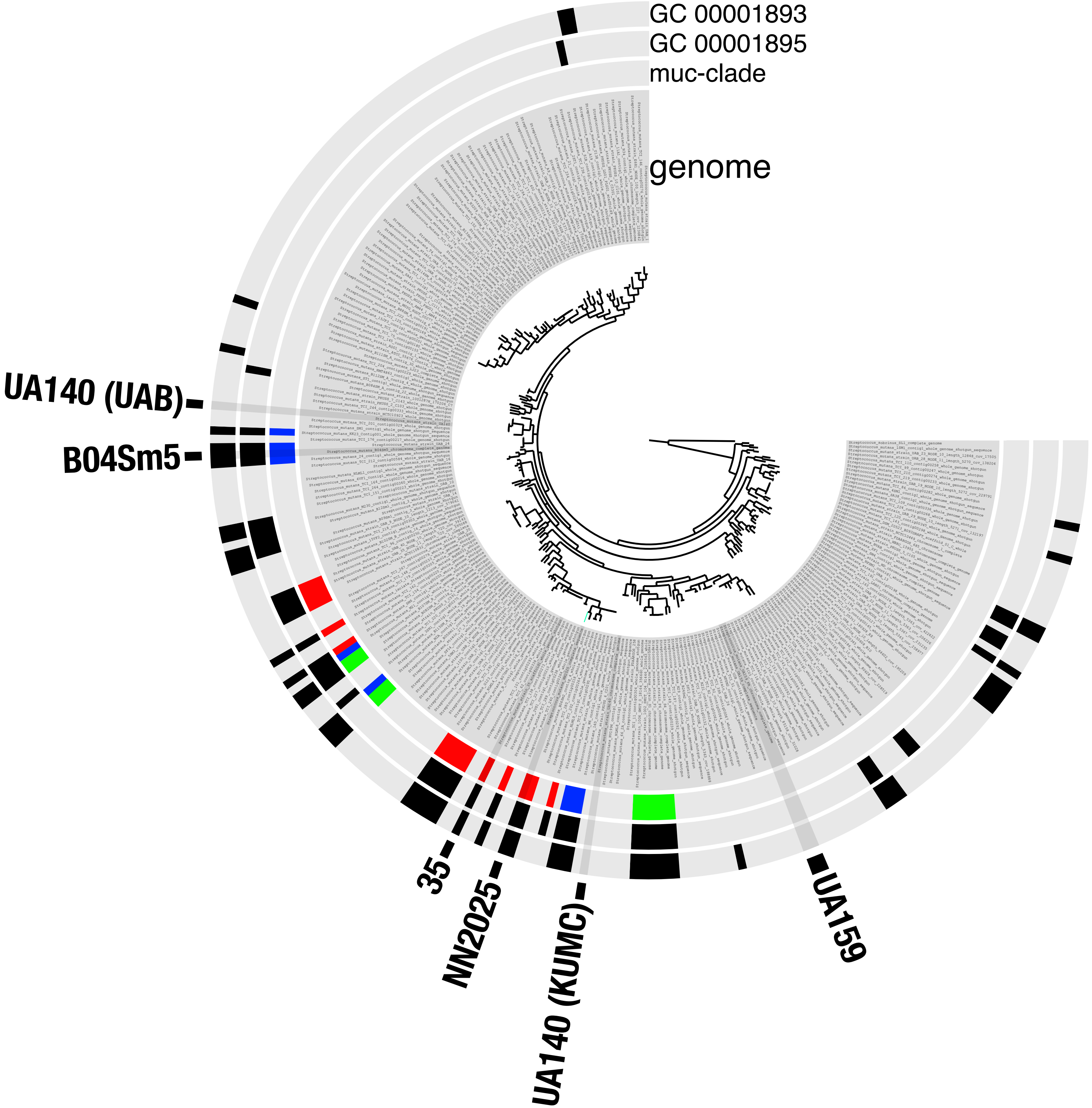
GC 00001893
GC 00001895
muc-clade
genome
UA140 (UAB)
B04Sm5
35
NN2025
UA140 (KUMC)
UA159

**Figure S4: Deletion of *mucG* or *mucH* has broad effects on the B04Sm5 transcriptome.** KEGG metabolic map of *S. mutans* metabolism (black nodes and edges) overlaid with the KOs exhibiting differential gene expression deletion of *mucG* (purple for decrease, yellow for increase), *mucH* (blue for decrease, orange for increase), or both (red for decrease, green for increase). Pathways of interest are indicated by labeled boxes, and genes of interest are indicated with labels.

**Figure S4**

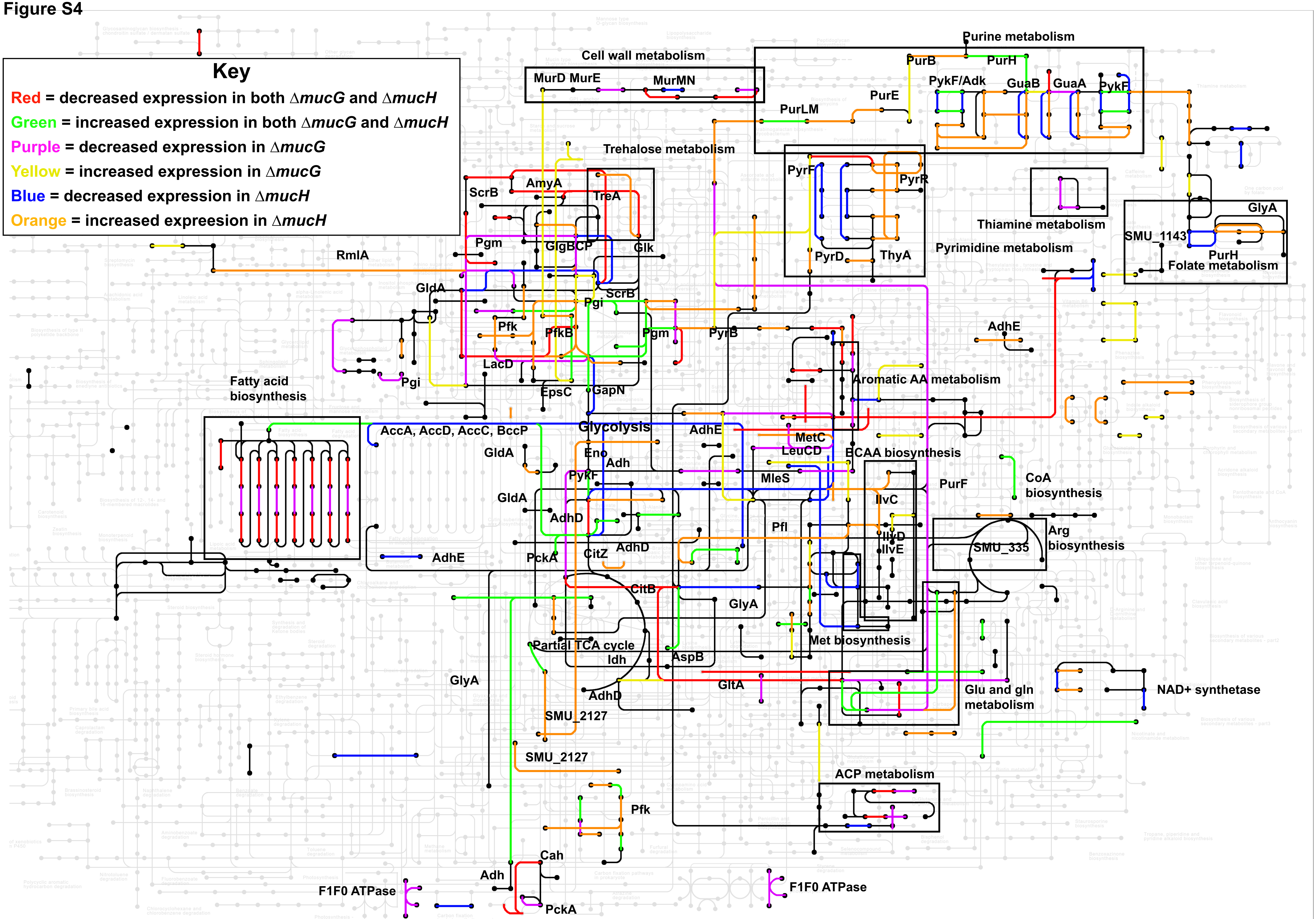
Key
Red = decreased expression in both ΔmucG and ΔmucH
Green = increased expression in both ΔmucG and ΔmucH
Purple = decreased expression in ΔmucG
Yellow = increased expression in ΔmucG
Blue = decreased expression in ΔmucH
Orange = increased expreesion in ΔmucH
Cell wall metabolism
MurD MurE
MurMN
Purine metabolism
PurB
PurH
PykF/Adk
GuaB
GuaA
PykF
PurE
PurLM
Trehalose metabolism
AmyA
ScrB
TreA
PyrF
PyrR
PyrD
ThyA
Thiamine metabolism
Pyrimidine metabolism
GlyA
SMU_1143
PurH
Folate metabolism
Pgm
GlgBCP
Glk
RmlA
GldA
ScrB
Pgi
Pfk
PfkB
Pgm
PyrB
AdhE
LacD
Pgi
Fatty acid biosynthesis
EpsC
GapN
Aromatic AA metabolism
AccA, AccD, AccC, BccP
Glycolysis
AdhE
MetC
LeuCD
BCAA biosynthesis
GldA
Eno
Adh
PykF
MleS
CoA biosynthesis
PurF
GldA
IlvC
AdhD
Pfl
IlvD
IlvE
Arg biosynthesis
SMU_335
AdhE
PckA
CitZ
AdhD
CitB
GlyA
Met biosynthesis
Partial TCA cycle
Idh
AspB
GlyA
GltA
AdhD
Glu and gln metabolism
NAD+ synthetase
SMU_2127
SMU_2127
ACP metabolism
Pfk
Cah
Adh
F1F0 ATPase
PckA
F1F0 ATPase
